# Three in One: A Case Report of Pulmonary Co-infection With a Virus, a Bacterium, and a Fungus in an Immunocompetent Adult

**DOI:** 10.7759/cureus.82025

**Published:** 2025-04-10

**Authors:** Ahmad B Al-Zughoul, Mohammed G Elhassan

**Affiliations:** 1 Internal Medicine, Saint Agnes Medical Center, Fresno, USA

**Keywords:** coccidioidomycosis, co-infection, coronavirus nl63, mycoplasma, pneumonia, valley fever

## Abstract

Pneumonia is a common cause of morbidity and mortality in the United States and worldwide. It is most commonly caused by a single organism, but co-infection can occur. Nevertheless, there are no or very few reported cases of triple acute co-infection with a virus, a bacterium, and a fungus in an immunocompetent patient without underlying chronic lung disease. Here, we discuss a case in which an otherwise healthy, elderly male exhibited a triple co-infection with coronavirus NL63, *Coccidioides immitis* or *Coccidioides posadasii* (also known as a cause of Valley fever), and *Mycoplasma pneumoniae*. We discuss the insight this clinical course can provide on future diagnosis and management of pulmonary coinfections.

## Introduction

Pneumonia is a common cause of morbidity and mortality in the United States and worldwide [1]. It is most commonly caused by a single organism. Nevertheless, pulmonary co-infection is not uncommon, especially among viruses and between a virus and a bacterium. It can occur in both pediatric and adult populations [2-4]. Most reports included pulmonary co-infections with viruses (e.g., respiratory syncytial virus, human rhinovirus, adenovirus, influenza and parainfluenza viruses, and severe acute respiratory syndrome coronavirus-2 (SARS‑CoV‑2)), bacteria (e.g., *Streptococcus pneumoniae, Haemophilus influenzae*, *Mycoplasma pneumoniae*, *Pseudomonas aeruginosa*, *Acinetobacter baumannii*, and *Stenotrophomonas maltophilia*), or fungi (e.g., *Aspergillus spp.*, *Pneumocystis spp.*, and *Rhizopus spp.*). To the best of our knowledge, there are no or very few reported cases of triple acute co-infection with a virus, a bacterium, and a fungus in an immunocompetent patient without underlying chronic or structural lung disease. Here, we discuss a case in which an otherwise healthy male exhibited a triple acute co-infection with coronavirus NL63, *Coccidioides*, and *Mycoplasma pneumoniae*, which necessitated hospitalization. We discuss the insight that this clinical course can provide on future diagnosis and management of pulmonary coinfections.

## Case presentation

**Figure 1:**
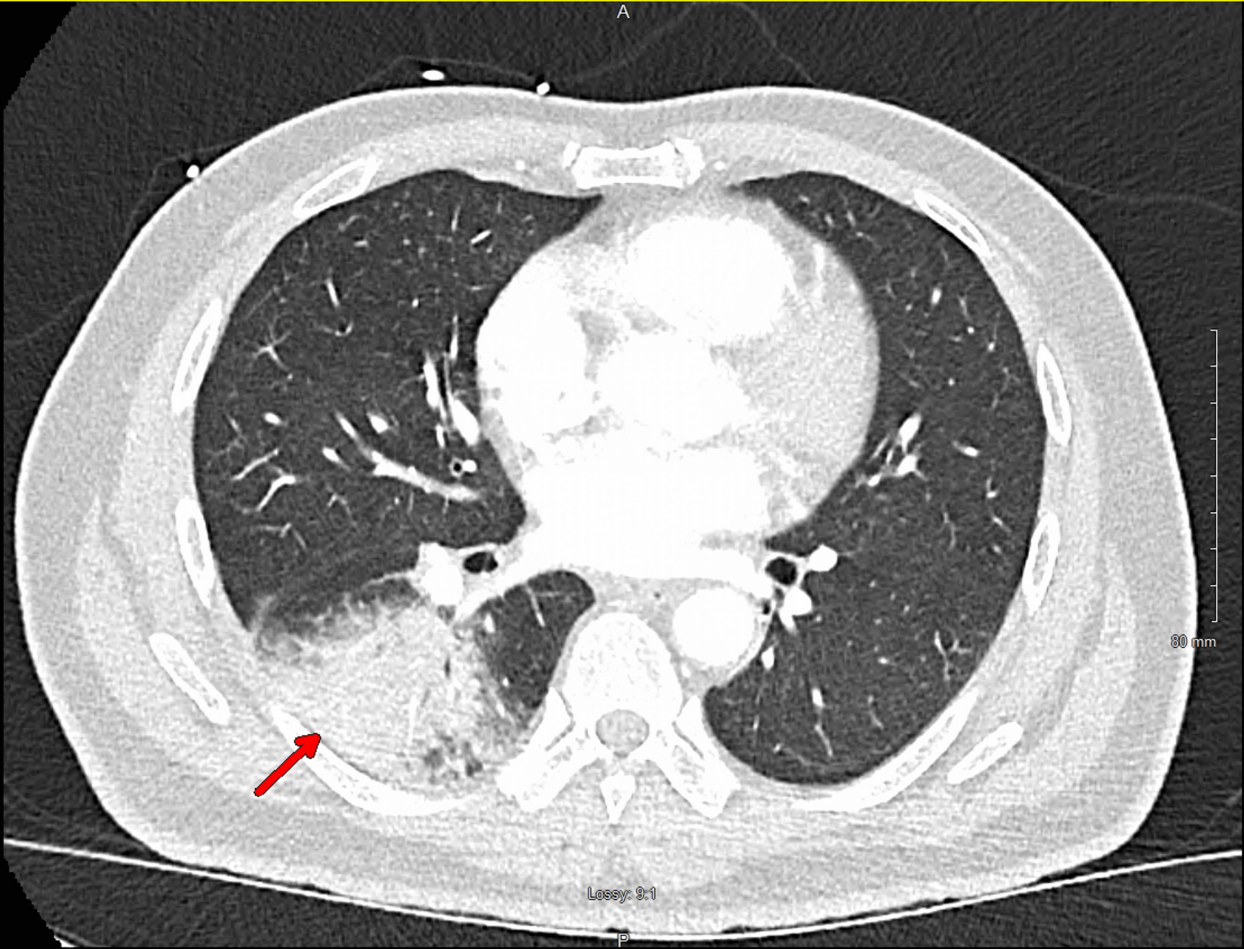
CT scan of the chest showing right lower lobe consolidation.

A 61-year-old male with no past medical history presented to the emergency department with complaints of skin rash, fever, shortness of breath, and cough for about a week. On admission, he was found to be febrile (38.8°C or 101.8°F) and tachycardic (115 bpm). Physical exam showed mild pharyngeal erythema and a few coarse crackles at the lung bases and a maculopapular rash on the trunk, shoulders, and upper thighs. Laboratory findings showed leukocytosis of 15.0 K/mcl (normal: 4.5-11.0 K/mcl) with neutrophilia at 12.22 K/mcl (normal: 2.6-8.2 K/mcl), and mild eosinophilia of 0.36 K/mcl (normal: 00.0-0.35 K/mcl). Chest X-ray showed a right lower lung infiltrate, confirmed with computed tomography (CT) of the chest as well, which did not show pleural effusion or lymphadenopathy (Figure 1). A multiplex polymerase chain reaction (PCR) respiratory panel called BioFire (originally from France but with headquarters in Salt Lake City, Utah) that screens for both common viral and bacterial pathogens was performed on a nasopharyngeal swab sample, and it was positive for *Mycoplasma pneumoniae* and coronavirus NL63. Because the patient lives in an area endemic of coccidioidomycosis, *Coccidioides spp.* immunoglobulin M (IgM) and immunoglobulin G (IgG) detected using enzyme-immunoassay (EIA) (manufactured by Premier, Inc., Charlotte, North Carolina) on the patient’s serum were requested on admission and came back negative.

The patient was admitted to the inpatient service and treated as community-acquired pneumonia (CAP) with azithromycin 500 milligrams IV daily and ceftriaxone 1 gram IV daily. After 48 hours of intravenous antibiotics, the patient remained symptomatic with cough and fever, and leukocytosis persisted, although the skin rash was improving. He was started on levofloxacin 750 mg IV daily to cover for the possibility of macrolide-resistant *Mycoplasma pneumoniae*. The patient was still having a cough and fever five days into admission, with a high total white blood count at 17.5 k/mcl, with increased eosinophilia at 1.08 K/mcl. Given a high clinical suspicion for acute pulmonary coccidioidomycosis, screening IgM and IgG antibodies with the same EIA were repeated, and both came back positive this time (Table 1). The quantitative test for IgG by complement fixation was negative. A culture of the lower respiratory tract with Gram stain was performed, which did not yield any significant results. Infectious disease consultation was requested, and the patient was started and discharged the following day on fluconazole 200 mg tablet by mouth daily for three months. Follow-up after discharge showed that the patient completed and tolerated the fluconazole course with resolution of his symptoms, and was reassured by his primary care physician that he does not need to extend the course of treatment.

**Table 1:** The results of the microbiological tests used to make the diagnosis of triple-pulmonary co-infection. Each column represents a day, with the first column on the right representing tests done on admission. PCR: polymerase chain reaction; RSV Ag: respiratory syncytial virus antigen; RT-PCR: reverse transcription-polymerase chain reaction.

BioFire respiratory panel	Day 1	Day 5
Adenovirus detection by PCR	Not detected	
Bordetella pertussis	Not detected	
Coronavirus 229E	Not detected	
Coronavirus HKU1	Not detected	
Coronavirus NL63	Detected	
Coronavirus OC43	Not detected	
Human metapneumovirus A and B	Not detected	
Influenza virus A PCR	Not detected	
Influenza virus B PCR	Not detected	
Monospot	Negative	
*Mycoplasma pneumoniae* by PCR	Detected	
Parainfluenza virus 1	Not detected	
Parainfluenza virus 2	Not detected	
Parainfluenza virus 3	Not detected	
Parainfluenza virus 4	Not detected	
RSV Ag	Not detected	
Respiratory virus panel molecular study		
Human rhinovirus/enterovirus	Not detected	
SARS-CoV-2 Qal RT-PCR	Not detected	
Enzyme-immunoassay		
Coccidioides IgG	Negative	Positive
Coccidioides IgM	Negative	Positive

## Discussion

The patient presented with typical clinical and radiological features of CAP, specifically lobar pneumonia, which usually suggests bacterial etiology [5]. *Mycoplasma pneumoniae* is a common cause of CAP, particularly in children and young adults. It usually presents as bilateral reticulonodular or patchy infiltrates, but can present as lobar pneumonia, and it can be associated with maculopapular rash among other non-pulmonary manifestations [6,7].

Non-COVID-19 coronavirus pneumonia presents with a variety of clinical and radiological features, including symptoms suggestive of bacterial pneumonia or flu-like symptoms commonly associated with SARS-CoV-2 or other viral infections. It typically shows mixed patterns on chest imaging, with lesions usually being bilateral and peripherally distributed. Coinfections can also be seen [8,9].

Coccidioidomycosis, commonly known as Valley fever, is a fungal infection caused by the dimorphic fungi *Coccidioides immitis *and *Coccidioides posadasii* that can present in acute, disseminated, or chronic forms, although many acquired infections are asymptomatic. Acute pulmonary involvement includes lobar or segmental consolidation, multifocal consolidation, and nodules on chest imaging with possible adenopathy and pulmonary effusions [10]. It can also manifest chronically with cavitary lesions or “coccidioidoma” and rarely as a disseminated disease with widespread pulmonary involvement [11].

PCR testing for respiratory pathogens is a sensitive and specific test and can be helpful clinically for rapid diagnosis and initiation of an appropriate management strategy for patients admitted with pneumonia. For example, the BioFire test showed a sensitivity of 98.1% and a specificity of 99.5% to 100% for detecting *Mycoplasma pneumoniae* when compared to reference laboratory-developed PCR assays [12,13]. And for detecting non-COVID-19 coronaviruses, it also showed a sensitivity of ≥75% and specificity of ≥87.2% [12,14,15].

The sensitivity and specificity of *Coccidioides* IgG and IgM antibodies using EIA for acute coccidioidomycosis pneumonia vary depending on the specific commercially available EIA kit used. For the MVista (manufactured by MiraVista Diagnostics, Indianapolis, Indiana) *Coccidioides* antibody detection EIA, the sensitivity for IgG antibody detection is 87.4%, and the specificity is 90%. For IgM antibody detection, the sensitivity is 61.2% and the specificity is 95.3%. For the IMMY Omega EIA (manufactured by IMMY, Norman, Oklahoma), the sensitivity for IgG antibody detection is 46.6%, and the specificity is 94.6%. For IgM antibody detection, the sensitivity is 22.3% and the specificity is 98.2%. For the Meridian Premier EIA (manufactured by Meridian Biosciences Inc., Cincinnati, Ohio), the sensitivity for IgG antibody detection is 70.9%, and the specificity is 96.4%. For IgM antibody detection, the sensitivity is 29.1% and the specificity is 99.1% [16].

Given the fact that the patient lives in an endemic area, in addition to his CT findings, skin rash, eosinophilia, and seroconversion, it was suspected that *Coccidioides* pneumonia played a larger role in the patient’s symptomatology than the other two pathogens detected by PCR, although it is difficult to ascertain that notion. Treatment of non-COVID-19 coronavirus pneumonia is largely supportive, and the patient received appropriate treatment for *Mycoplasma pneumoniae* without resolution of symptoms, fever, or leukocytosis. Although the screening serological tests for coccidioidomycosis using EIA can be associated with false-positive cases because they have high sensitivity but less specificity, the seroconversion and the clinical presentation both support true positivity and actual infection [17]. The quantitative test for IgG by complement fixation was negative, but it is reported by data from a laboratory experienced in coccidioidomycosis testing, where our hospital sends all positive results to, that about 15% of patients with serologically confirmed Valley fever never develop a detectable complement fixation titer. Moreover, complement fixation tests result as positive later in the course of the disease, compared to the EIA tests, which can be detected within about a week of the onset of symptoms [18].

## Conclusions

This case report sheds light on how the presence of multiple respiratory pathogens can potentially complicate the clinical course and management of pneumonia in such patients. Clinicians should be aware of the sensitivity and specificity of microbiological tests used to diagnose specific respiratory pathogens in their institution to make informed management decisions for their patients. Consultation with infectious disease specialists can be very helpful as well in complicated or difficult cases or cases not responding to appropriate management.
